# Selective inhibition of yeast regulons by daunorubicin: A transcriptome-wide analysis

**DOI:** 10.1186/1471-2164-9-358

**Published:** 2008-07-30

**Authors:** Marta Rojas, Marta Casado, José Portugal, Benjamin Piña

**Affiliations:** 1 Institut for Molecular Biology of Barcelona, IBMB-CSIC, Jordi Girona, 18. 08034 Barcelona, Spain

## Abstract

**Background:**

The antitumor drug daunorubicin exerts some of its cytotoxic effects by binding to DNA and inhibiting the transcription of different genes. We analysed this effect *in vivo *at the transcriptome level using the budding yeast *Saccharomyces cerevisiae *as a model and sublethal (IC_40_) concentrations of the drug to minimise general toxic effects.

**Results:**

Daunorubicin affected a minor proportion (14%) of the yeast transcriptome, increasing the expression of 195 genes and reducing expression of 280 genes. Daunorubicin down-regulated genes included essentially all genes involved in the glycolytic pathway, the tricarboxylic acid cycle and alcohol metabolism, whereas transcription of ribosomal protein genes was not affected or even slightly increased. This pattern is consistent with a specific inhibition of glucose usage in treated cells, with only minor effects on proliferation or other basic cell functions. Analysis of promoters of down-regulated genes showed that they belong to a limited number of transcriptional regulatory units (regulons). Consistently, data mining showed that daunorubicin-induced changes in expression patterns were similar to those observed in yeast strains deleted for some transcription factors functionally related to the glycolysis and/or the cAMP regulatory pathway, which appeared to be particularly sensitive to daunorubicin.

**Conclusion:**

The effects of daunorubicin treatment on the yeast transcriptome are consistent with a model in which this drug impairs binding of different transcription factors by competing for their DNA binding sequences, therefore limiting their effectiveness and affecting the corresponding regulatory networks. This proposed mechanism might have broad therapeutic implications against cancer cells growing under hypoxic conditions.

## Background

Understanding the mode of action of antitumor drugs is considered an absolute prerequisite for the advancement on the design of new drugs. It is generally believed that antitumor activity is mediated by the capacity of certain drugs to induce DNA damage and trigger apoptosis. However, there are many indications that this mechanism, whatever relevant may it be, does not account for all therapeutic effects of some antitumor drugs [1,2].

The anthracycline antibiotic daunorubicin is widely used in cancer chemotherapy [3]. It accumulates in the nuclei of living cells and intercalates into DNA quantitatively [4,5], a property associated to some of the most relevant effects of the drug: inhibition of DNA replication and gene transcription [1,6,7], displacement of protein factors from the transcription complex [8] and topoisomerase II poisoning [9]. Daunorubicin has the property of arresting cell growth at drug concentrations not sufficient for promoting noticeable DNA damage, and through mechanisms that differ from the apoptotic pathway [7]. These findings impelled to define new mechanisms of daunorubicin antiproliferative activity at clinically relevant concentrations.

Daunorubicin shows remarkable sequence specificity for 5'-WCG-3' DNA tracts [10]. This property has led to the suggestion that daunorubicin may compete with transcription factors with overlapping recognition sites for binding to DNA. This model would explain several effects of daunorubicin, such as inhibition of RNA polymerase II [1,6,7] and the suppression of the co-ordinate initiation of DNA replication in *Xenopus *oocyte extracts [11].

To test the capacity of daunorubicin to displace key transcription factors from their binding sites in chromatin *in vivo*, and, therefore, to inhibit their action [6], we used the yeast *Saccharomyces cerevisiae *as a model. In a previous work [12], we showed that yeast strains deficient in ergosterol synthesis *(Δerg6 *strains) are particularly sensitive to daunorubicin, overcoming one of the main setbacks to the use of yeast in pharmacological studies, which is their resistance to many anti-tumour drugs [13,14].

We demonstrated that daunorubicin treatment in Δ*erg6 *cells precluded activation of several genes required for galactose utilization (GAL genes) and, consequently, treated cells were unable to growth in galactose. This effect was related to the presence of CpG steps in the cognate DNA binding sequence of Gal4p, the key transcription factor for activation of GAL genes [12,15]. The present work aims to extend this type of analysis to the totality of the yeast genome, in order to assess the generality of this model.

## Results

### Effects of daunorubicin on the yeast transcriptome

**Figure 1:**
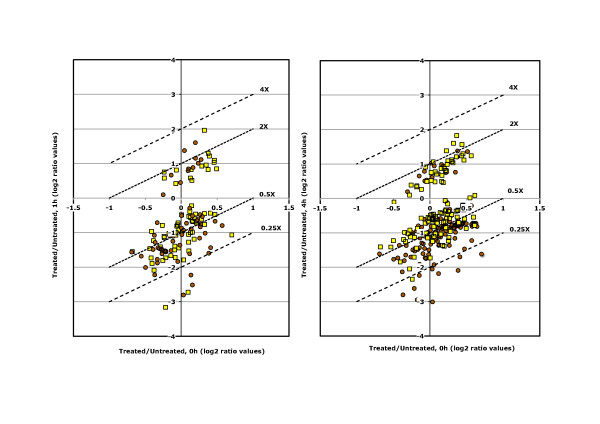
**Effects of daunorubicin to the yeast transcriptome.** Expression data from treated and untreated cells (expressed as binary logs) were compared before and after one and four hours of incubation with daunorubicin. Data are represented as log_2 _of the ratios of gene expression values after 1 h (left) and 4 h (right) of daunorubicin treatment versus the initial values (Time 0). Only genes whose expression was significantly altered by the treatment (T-test, brown dots, p < 10^-5^, yellow squares, p < 10^-2^) are shown. Discontinuous lines in the plots indicate the calculated positions of genes changed by 4-, 2-, 0.5- and 0.25-fold; they are included as references to compare with the changes in expression of different genes.

**Figure 2:**
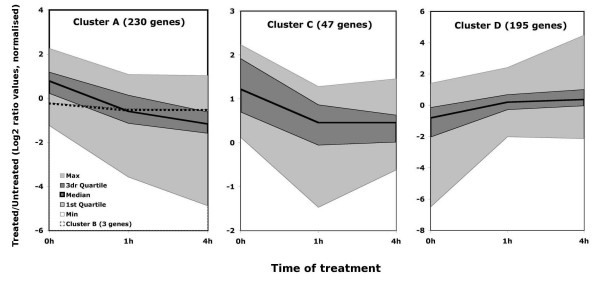
**Transcriptional profiles for genes classified into clusters by SOM.** Data are shown as logarithmic values of the ratio of fluorescence between treated and untreated cells before (0 h) and 1 and 4 hours after treatment. No correction was performed to compensate differences in labelling or detection of the two fluorochromes. The thick solid lines in the middle of the graphs correspond to median values, coloured areas correspond to the intervals between 1st and 3rd quartiles (dark gray) and the total distribution (light gray). Averaged values for Cluster B (3 genes, discontinuous line) in included in the Cluster A plot.

The effects of daunorubicin on the yeast transcriptome were studied after 1 h and 4 h of treatment (Figure 1). The results indicate a general inhibitory effect of daunorubicin at both time points, as down regulated genes predominate over up regulated ones, and this trend was especially significant when considering genes whose expression changed by more than four-fold (lines "4X" and "0.25X" in Figure 1). Multi-array analysis of the expression changes in the whole dataset confirmed these trends. ANOVA analysis of normalized data showed statistically significant differences in expression upon daunorubicin treatment for 475 genes (14%) at least in one of the time points analysed. Affected genes were grouped in four clusters by a Self-Organising Maps (SOM) algorithm, according to their differential expression at the three time points analysed (Figure 2, list of genes for each cluster in Table 1). Clusters A to C (280 genes in total) corresponded to genes whose transcription decreased upon daunorubicin treatment, whereas all genes that became activated by the treatment (195 genes) were grouped in Cluster D. Genes in Clusters C and D showed very little or no difference in expression between one and four hours of treatment (see the horizontal median line in the corresponding plots between time points 1 h and 4 h in Figure 2), whereas genes in Cluster A were the only ones in which the effect (an inhibition, in this case) after four hours of treatment was significantly stronger than the observed after one hour (Figure 2). Cluster B, consisting only in three genes, was the only one in which the effect was stronger at one hour than at four hours. Our data thus indicated that most daunorubicin-related changes in gene expression were already significant after only one hour of treatment and that these effects either increased or remained stable after four hours for essentially all analysed genes.

**Table 1:** Gene clusters defined by SOM analysis

Cluster A				Cluster B	Cluster C	Cluster D			
			
*AAH1*	*GPI12*	*PPM1*	*YDR428C*	*URA2*	*ACT1*	*ACC1*	*RPC31*	*YBL051C*	*YMR074C*
*AAT2*	*GPM1*	*PRB1*	*YDR453C*	*YJU3*	*ARG8*	*ANB1*	*RPC40*	*YBL057C*	*YMR085W*
*ACO1*	*GPM2*	*PRY1*	*YDR516C*	*YML056C*	*ARO4*	*ARL1*	*RPG1*	*YBR012W-B*	*YMR130W*
*ADE12*	*GRE2*	*PRY3*	*YDR539W*		*AYR1*	*BFR1*	*RPL13B*	*YCL019W*	*YMR158C-B*
*ADE17*	*GRE3*	*PSA1*	*YFR017C*		*CAR2*	*CAF20*	*RPL32*	*YCR082W*	*YNL054W-B*
*ADH1*	*GSF2*	*PST1*	*YGL121C*		*CDC91*	*CBF5*	*RPL34B*	*YDL076C*	*YNL296W*
*ADH2*	*GSY2*	*RAD51*	*YGL157W*		*DAK1*	*CCT5*	*RPL6A*	*YDL157C*	*YNR046W*
*ADH5*	*GTT1*	*RHR2*	*YGP1*		*ERG10*	*CDC20*	*RPL6B*	*YDL166C*	*YOL026C*
*ALD4*	*GYP7*	*RIB1*	*YGR045C*		*FAS1*	*CDC33*	*RPN10*	*YDR034C-D*	*YOL092W*
*ALD6*	*HHO1*	*RIB4*	*YGR161C*		*GDH1*	*CDC60*	*RPO26*	*YDR060W*	*YOL124C*
*AMS1*	*HMT1*	*RIP1*	*YHL021C*		*GLT1*	*COP1*	*RPS11B*	*YDR084C*	*YOR021C*
*ARA1*	*HOR2*	*RME1*	*YHM1*		*NUP82*	*CPR6*	*RPS19A*	*YDR098C-B*	*YOR262W*
*ARG1*	*HSP104*	*RNR1*	*YHR087W*		*PFK1*	*DIB1*	*RPS26A*	*YDR154C*	*YOR343C-A*
*ARG4*	*HSP12*	*SCM4*	*YIL011W*		*PHB1*	*DPB4*	*RPS4B*	*YDR210C-D*	*YOR343C-B*
*ARG5*	*HSP26*	*SCS7*	*YIL056W*		*PYC2*	*DST1*	*RPS8A*	*YDR210W-D*	*YOR382W*
*ARO3*	*HSP42*	*SCW11*	*YIL077C*		*QCR10*	*FCY1*	*RPT3*	*YDR261C-D*	*YPL199C*
*ASH1*	*HXK1*	*SDS24*	*YJL016W*		*QCR2*	*FKB2*	*RRP4*	*YDR261W-B*	*YPL225W*
*BAP2*	*HXK2*	*SGE1*	*YJL094C*		*RFC5*	*FPR1*	*RRP5*	*YDR316W-B*	*YPR137C-B*
*BAP3*	*HXT1*	*SHM2*	*YJR008W*		*RNR4*	*FRQ1*	*RRP9*	*YDR361C*	*YPR158W-B*
*BAT2*	*HXT2*	*SNO1*	*YKL151C*		*STI1*	*HCH1*	*RRS1*	*YDR365W-B*	*YPS7*
*CAP2*	*IDH1*	*SNQ2*	*YKR067W*		*STP3*	*HIR1*	*RSC6*	*YDR449C*	*YPT31*
*CBP4*	*IDH2*	*SNZ1*	*YLL012W*		*TEF1*	*HIS7*	*RVB2*	*YER007C-A*	
*CHA1*	*ILV5*	*SPI1*	*YLR110C*		*TKL1*	*HRP1*	*SAS10*	*YER092W*	
*CHS1*	*INO1*	*SRL3*	*YLR111W*		*TSA1*	*HRR25*	*SBH1*	*YER126C*	
*CIT1*	*IPT1*	*SRY1*	*YLR122C*		*TTR1*	*HRT1*	*SEC21*	*YER138C*	
*CLN2*	*IRA2*	*SSA1*	*YLR231C*		*UGA1*	*ILS1*	*SEC65*	*YER160C*	
*COQ1*	*KNS1*	*SSA2*	*YLR331C*		*URA4*	*IMP4*	*SEC72*	*YER183C*	
*COS1*	*LAP4*	*SSD1*	*YLR352W*		*YBR070C*	*KAP123*	*SER3*	*YFH1*	
*COS7*	*LSC2*	*SUN4*	*YLR414C*		*YDR214W*	*KRI1*	*SES1*	*YFL002W-A*	
*COX20*	*MCR1*	*TAT2*	*YLR454W*		*YDR476C*	*KRR1*	*SIT1*	*YFL004W*	
*CPA1*	*MDH1*	*TDH1*	*YML128C*		*YER134C*	*LOS1*	*SKP1*	*YGR038C-B*	
*CTS1*	*MDH2*	*TDH2*	*YMR090W*		*YER182W*	*LYS7*	*SMD3*	*YGR081C*	
*CYC3*	*MEP1*	*TDH3*	*YMR173W-A*		*YGL047W*	*MGM101*	*SNF8*	*YGR161W-B*	
*CYT1*	*MEP3*	*THO1*	*YMR181C*		*YGR201C*	*NAT3*	*SNT309*	*YHR052W*	
*DDR2*	*MET6*	*TIR2*	*YMR315W*		*YHR049W*	*NIP7*	*SPB1*	*YHR214C-B*	
*DDR48*	*MMD1*	*TPI1*	*YNL200C*		*YIL087C*	*NMD3*	*SPE3*	*YHR214C-C*	
*DED1*	*MOG1*	*TPS2*	*YNL212W*		*YIR035C*	*NOP12*	*SPE4*	*YIL127C*	
*DYN1*	*MRPL35*	*TRR2*	*YOL101C*		*YLL023C*	*NOP58*	*SSF1*	*YJR027W*	
*EHT1*	*MSF1'*	*TSL1*	*YOR009W*		*YLR112W*	*NPI46*	*SSP120*	*YJR029W*	
*ENO1*	*MTF2*	*TUF1*	*YOR022C*		*YLR356W*	*NPT1*	*STS1*	*YKL014C*	
*ENO2*	*NCE102*	*UGP1*	*YOR062C*		*YMR178W*	*NRD1*	*SUI1*	*YKL054C*	
*ERG11*	*NCR1*	*URA1*	*YOR081C*		*YNL100W*	*OLI1*	*SUI2*	*YKR081C*	
*ERG26*	*OAC1*	*UTR2*	*YOR258W*		*YNL305C*	*OST3*	*SXM1*	*YKT6*	
*ERG5*	*OPI3*	*VAP1*	*YOR280C*		*YPL101W*	*PCL1*	*TIF11*	*YLR009W*	
*ERG6*	*PBI2*	*VID24*	*YOR289W*		*YPR098C*	*PFS2*	*TIF34*	*YLR035C-A*	
*EXG1*	*PCL7*	*YAL053W*	*YOR338W*		*YSA1*	*PHO11*	*TIF35*	*YLR065C*	
*FBA1*	*PDC1*	*YBL049W*	*YPL004C*			*PHO12*	*TIP1*	*YLR106C*	
*FUN14*	*PDC5*	*YBL064C*	*YPL066W*			*PRE10*	*TPM1*	*YLR157C-B*	
*GCV1*	*PDH1*	*YBR006W*	*YPL134C*			*PRE2*	*TPM2*	*YLR159W*	
*GCV2*	*PDR5*	*YBR053C*	*YPL156C*			*PRE3*	*TRP1*	*YLR221C*	
*GCY1*	*PET8*	*YBR230C*	*YPR153W*			*PRE9*	*UBA1*	*YLR227W-B*	
*GLK1*	*PEX11*	*YDC1*	*YPR172W*			*PUP2*	*UBC1*	*YLR410W-B*	
*GLO1*	*PGK1*	*YDL124W*	*YRA1*			*RDI1*	*UBC13*	*YML039W*	
*GLY1*	*PGM2*	*YDR041W*	*YTP1*			*RLP7*	*UBC4*	*YML093W*	
*GND1*	*PHO3*	*YDR233C*	*ZRT1*			*RNA14*	*UBC6*	*YML125C*	
*GPA2*	*PIR1*	*YDR319C*	*ZRT2*			*RNH70*	*URA5*	*YMR045C*	
*GPD2*	*PLB1*	*YDR387C*				*RPA49*	*VAR1*	*YMR046W-A*	
*GPH1*	*PPA2*	*YDR391C*				*RPC10*	*YBL005W-B*	*YMR050C*	

**Figure 3:**
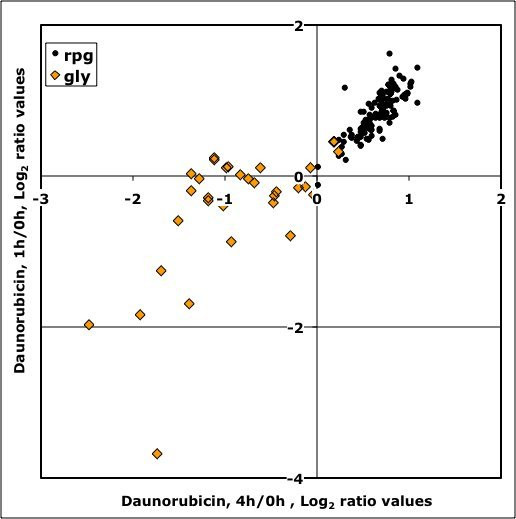
**Transcriptional rate changes for Ribosomal Protein genes (solid dots) and Glycolytic genes (diamonds) after 1 (Y-axis) and 4 h (X-axis) of daunorubicin treatment.** Data are expressed as logarithmic values of expression ratios between treated and untreated cells.

**Figure 4:**
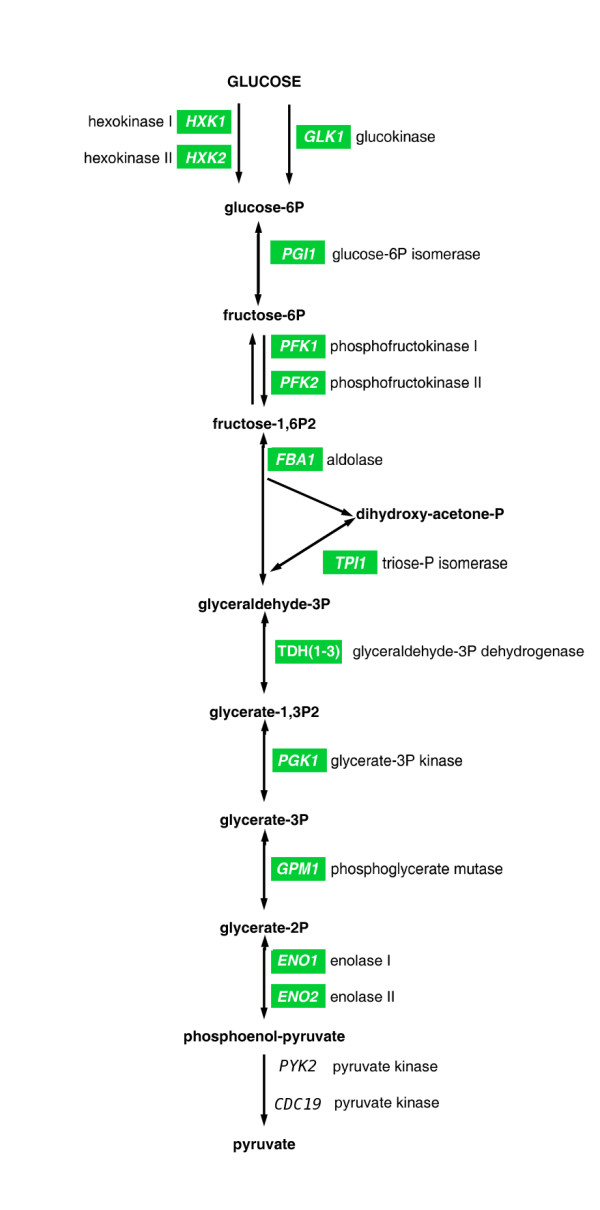
**Scheme of the glycolytic pathway.** Genes codifying for the enzymes implicated in each step are detailed; green labels indicate genes whose expression was reduced upon daunorubicin treatment.

Gene Ontology (GO) analysis of genes activated and repressed by daunorubicin treatment showed a very different distribution of GO categories for both groups. Up-regulated genes fell into three main functional categories: Genes related to ribosome assembly and metabolism, Ty transposition, and proteolytic processes (Table 2). Whereas the two last categories may indicate a certain level of stress, up regulation of ribosome assembling-related genes usually correlates with a positive effect in cell growth. In contrast, GO analysis of genes down regulated by daunorubicin showed a general decrease of energy-producing metabolism, including genes involved in fermentation and in the tricarboxylic acid cycle. A significant proportion of down-regulated genes appeared involved in the metabolism of nitrogen compounds, including amino acids (Table 3). The dissociation between expression of ribosomal and glycolytic genes upon daunorubicin treatment can be observed in Figure 3, which shows up-regulation of most ribosomal protein genes and down-regulation of sugar and alcohol-metabolism related genes at one and four hours of daunorubicin treatment. Figure 4 shows a scheme of the glycolytic pathway, highlighting genes down regulated by daunorubicin. These genes codify the enzymes responsible for no less than 9 consecutive steps of the pathway. Therefore, the data suggests that the fermentation capacity should be depressed in daunorubicin-treated yeast cells.

**Table 2:** GO Term finder results for genes up-regulated by daunorubicin

**Gen Ontology Term clustering**			
Functional categories	GOID	GOID- associated functions	
	
A	32196; 32197	Transposition, Ty metabolism	
B	27; 460; 466; 6364; 6396; 6996; 16043; 16070; 16072; 22613; 22618; 42254; 42255; 42257; 42273; 43170; 65003	Ribosome assembling (Protein and rRNA) Proteolysis. Ubiquitin-	
C	6508; 6511; 19941; 30163; 43632; 44257; 51603	mediated preoteolysis.	

**Gene Clustering**			

Distribution among functional categories	Genes	Main gene functions	Number of genes

A only	FCY1; FRQ1; HIS7; PCL1; PHO11; SER3; SIT1; SPE3; SPE4; TRP1; URA5; YBL005W-B; YBR012W-B; YCL019W; YDR034C-D; YDR098C-B; YDR210C-D; YDR261C-D; YDR261W-B; YDR316W-B; YDR365W-B; YER138C; YER160C; YFL002W-A; YGR038C-B; YGR161W-B; YHR214C-B; YHR214C-C; YJR027W; YJR029W; YLR035C A; YLR157C-B; YLR227W-B; YLR410W-B; YML039W; YMR045C; YMR050C; YNL054W-B; YPR137C-B; YPR158W-B	Ty genes	40
B only	ACC1; ANB1; ARL1; BFR1; CAF20; CBF5; CCT5; CDC33; CDC60; COP1; CPR6; DIB1; DPB4; DST1; FPR1; HCH1; HIR1; HRP1; HRR25; ILS1; IMP4; KAP123; KRI1; KRR1; LOS1; MGM101; NAT3; NIP7; NMD3; NOP12; NOP58; NPT1; NRD1; OST3; PFS2; RDI1; RLP7; RNA14; RNH70; RPA49; RPC10; RPC31; RPC40; RPG1; RPL13B; RPL32; RPL34B; RPL6A; RPL6B; RPO26; RPS11B; RPS19A; RPS26A; RPS4B; RPS8A; RRP4; RRP5; RRP9; RRS1; RSC6; RVB2; SAS10; SEC21; SEC65; SEC72; SES1; SMD3; SNT309; SPB1; SSF1; SSP120; SUI1; SUI2; SXM1; TIF11; TIF34; TIF35; TIP1; TPM1; TPM2; UBA1; UBC13; YFH1; YIL127C; YKT6; YNL296W; YOR021C; YPT31	Ribosomal protein genes, rRNA metabolism, translation.	87
C>B	CDC20; HRT1; PRE10; PRE2; PRE3; PRE9; PUP2; RPN10; RPT3; SKP1; SNF8; STS1; UBC1; UBC4; UBC6	Endopeptidases, ubiquitin-protein ligases	15

No GO Term			53

**Table 3:** GO Term finder results for genes down-regulated by daunorubicin

**Gen Ontology Term clustering**			
Functional categories	GOID	GOID- associated functions	
	
A	5975; 5996; 6006; 6007; 6066; 6067; 6082; 6090; 6094; 6096; 6113; 6766; 6767; 9056; 9063; 15980; 16051; 16052; 19318; 19319; 19320; 19752; 32787; 44248; 44262; 44275; 46164; 46165; 46364; 46365;	Alcohol and carbohydrate metabolism (including glycolysis). Vitamin and organic acid metabolism.	
B	6091; 6099; 6100; 6519; 6520; 6536; 6537; 6807; 8652; 9064; 9084; 9308; 9309; 44271; 46356	Amino acid metabolic process. Tricarboxilic acid cycle.	

**Gene Clustering**			

Distribution among functional categories	Genes	Main gene functions	Number of genes

A>>B	GPD2; PDC1; PDC5; PCL7; UGP1; DAK1; GLO1; INO1; PGM2; MDH2; PSA1; GRE3; GCY1; GLK1; TPI1; HXK1; HXK2; PFK1; VID24; GND1; TKL1; PYC2; PGK1; TDH3; ENO1; ENO2; TDH1; TDH2; FBA1; GPM1	Glycolysis	30
A>B	AAH1; ADH1; ADH2; ADH5; ALD4; ALD6; AMS1; ARA1; AYR1; CTS1; EHT1; ERG10; ERG11; ERG26; ERG5; EXG1; FAS1; GPH1; GSY2; HOR2; LAP4; MDH1; PDH1; PEX11; PHO3; PRB1; RHR2; RIB1; RIB4; SCS7; SNO1; SNZ1; TPS2; TSL1	Alcohol, lipid and sterol metabolism	34
A ≈ B	AAT2; BAT2; CAR2; CHA1; COX20; GCV1; GCV2; GLY1; LSC2; MCR1; PPA2; QCR10; QCR2; RIP1; SRY1; UGA1	Amino acid metabolism. Respiration	16
A<B	ACO1; ARG1; ARG4; ARG8; ARO3; ARO4; CIT1; CPA1; CYT1; GDH1; GLT1; IDH1; IDH2; ILV5; MEP1; MEP3; MET6; URA2	Nitrogen compound (including amino acids) metabolism. Tricarboxilic acid cylce	18

No GO term			181

The effects of daunorubicin treatment in gene expression of 15 selected genes were validated by qRT-PCR (list of genes and primers in Table 4, results in Table 5). The results, presented as ratios between treated and untreated cells at 0 h and 4 h of treatment, include data from up to 5 biological replicates, showed a general good agreement with microarray data. Most (8 out of 9) sugar and alcohol-metabolism related genes showed a 2 to 4 fold decrease on expression of after 4 h of treatment, a behaviour comparable to the one observed in the microarray analysis. Similarly, two out of the three amino acid metabolism genes analysed showed a 3 to 4 fold decrease on expression. In contrast, a small, but significant, increase on the expression of the ribosomal protein genes *RPS28A *was also observed, also in agreement with the general trend observed for ribosomal-protein genes in the microarray data. We added to this analysis the heat-shock protein *HSP26*, as a representative of a small group of HSP genes (*HSP12*, *HSP26*, *HSP42 *and *HSP104*) with appeared down regulated by daunorubicin in the microarray analysis (Table 1). These results were corroborated by qRT-PCR quantitation, which showed 8-fold reduction of HSP26 transcription after four hours of daunorubicin treatment (Table 5). These results confirmed the general decrease in genes related with glucose utilisation while transcription of ribosomal protein gene was either not affected or slightly increased.

**Table 4:** Primers used in this study

**GENE**	**Primer Sequence**	**Function**
*ACO1*	for: 5'-GTGGTGCTGATGCCGTTG-3'	Aconitase
	rev: 5'-CCTTCAATTCCCATGGACGA-3'	
*ACT1*	for: 5'-TGTGTAAAGCCGGTTTTGCC-3'	Actin
	rev: 5'-TTGACCCATACCGACCATGAT-3'	
*ARG1*	for: 5'-GCCCACATTTCTTACGAGGC-3'	Arginosuccinate synthetase
	rev: 5'-TGGTCCGGAGCATCCATT-3'	
*ARG4*	for: 5'-AAATTTGTCCGTCATCCAAACG-3'	Argininosuccinate lyase
	rev: 5'-CCGGTGTGGACTTTACCAGC-3'	
*CAR2*	for: 5'-CATCGCCCAATTGAAAGCTC-3'	L-ornithine transaminase
	rev: 5'-CCTTGGATGGGTCGATTACG-3'	
*CDC19*	for: 5'-TGGCCATTGCTTTGGACAC-3'	Pyruvate kinase
	rev: 5'-GGTGAAGATCATTTCGTGGTTTG-3'	
*FBA1*	for: 5'-AATGCTTCCATCAAGGGTGC-3'	Fructose 1,6-bisphosphate aldolase
	rev: 5'-CAACTGGGATACCGTAAGCTG-3'	
*GPM1*	for: 5'-TCACCGGTTGGGTTGATGTTA-3'	Glycerate Phosphomutase
	rev: 5'-TCCTTCAACAATTCACCGGC-3'	
*HSP26*	for: 5'-AGAGGCTACGCACCAAGACG-3'	Heat Shock Protein
	rev: 5'-AGAATCCTTTGCGGGTGTGT-3'	
*HXK1*	for: 5'-GTTGACAGCGAGACCTTGAGAA-3'	Hexokinase isoenzyme 1
	rev: 5'-CAACCGGGAATCATTGGAAT-3'	
*PGI1*	for: 5'-CTCAAAGAACTTGGTCAACGAT-3'	Phosphoglucoisomerase
	rev: 5'-CAAACCGGTGACGTTAGCCT-3'	
*PGK1*	for: 5'-CCCAGGTTCCGTTCTTTTGTTG-3'	3-phosphoglycerate kinase
	rev: 5'-TTGACCATCGACCTTTCTGGA-3'	
*RPO21*	for: 5'-AGGTTTGCTGCAATTTGGACTT-3'	RNA polymerase II largest subunit B220
	rev: 5'-CAACCTCCCCTTGATACGAGC-3'	
*RPS28A*	for: 5'-AGCCAAGGTCATCAAAGTTTTAGG-3'	Ribosomal Protein of the Small subunit
	rev: 5'-TTCCAAGAATTCGACACGGAC	
*TDH(1-3)*	for: 5'-AGACTGTTGACGGTCCATCCC-3'	Glyceraldehyde-3-phosphate dehydrogenase
	rev: 5'-AAGCGGTTCTACCACCTCTCC-3'	
*HOR2*	for: 5'-GTGCAACGCTTTGAACGCT-3'	Glicerol-1-phosphatase
	rev: 5'-GAAGTTGCCACAGCCCATTT-3'	
*TPS2*	for: 5'-TCATGCCCCATGGCCTAGTA-3'	Trehalose-6-phosphate phosphatase
	rev: 5'-TTTCTACGTGGCAAACAACGAA-3'	
*GLO1*	for: 5'-AGGATCCAGCAAGGACCGTT-3'	Glyoxalase
	rev: 5'-GCTTCATACCGAAGTGTTCGG-3'	

**Table 5:** Differential expression in daunorubicin-treated versus non-treated cells, measured by RT-qPCR

	Treated/Non treated^a)^							
					
Function	ORF	Time 0	Time 4 h	Fold variation (4 h/0 h)	*p*^*b*)^	Corrected *p * (Bonferroni)	*n * (technical replicates)	*n * (biological replicates)
	*ACO1*	0.001	-1.090	0.470	0.001	0.020	60	5
	*CDC19*	0.034	-1.132	0.446	6.3 × 10^-13^	9.5 × 10^-12^	108	5
	*FBA1*	0.005	-1.207	0.432	1.0 × 10^-13^	1.5 × 10^-12^	60	5
	*GPM1*	-0.005	-0.948	0.520	3.0 × 10^-8^	4.5 × 10^-7^	60	5
Energy metabolism	*HOR2*	-0.010	-1.413	0.378	9.0 × 10^-4^	0.014	24	2
	*HXK1*	0.315	-1.935	0.210	1.6 × 10^-21^	2.5 × 10^-20^	72	5
	*PGI1*	0.005	-0.061	0.956	0.80	> 0.05	60	5
	*PGK1*	0.005	-1.228	0.425	8.9 × 10^-19^	1.3 × 10^-17^	60	5
	*TDH*	-0.015	-1.428	0.375	5.9 × 10^-12^	8.8 × 10^-11^	60	5

	*ARG1*	-0.010	-2.032	0.246	2.1 × 10^-7^	3.2 × 10^-6^	24	2
Amino acid metabolism	*ARG4*	-0.001	-1.413	0.376	5.3 × 10^-6^	8.0 × 10^-5^	23	2
	*CAR2*	-0.011	-0.294	0.822	0.09	> 0.05	35	3

	*ACT1*	-0.480	-1.440	0.514	0.126	> 0.05	8	3
Others	*HSP26*	0.081	-2.921	0.125	5.1 × 10^-8^	7.6 × 10^-7^	24	2
	*RPS28A*	-0.005	0.476	1.396	0.002	0.028	60	5
	*TPS2*	0.002	0.120	1.086	0.42	> 0.05	22	2

a) Data expressed as dual logarithmic values of expression ratios, treated versus untreated. Corrected by *RPO21 *expression.

b) Student's T-Test, time 0 *versus *time 4 h ratios

### Identification of transcription factors associated to daunorubicin-repressed genes

Transcription factors reported to bind to the promoters of daunorubicin-repressed genes were identified using the on-line bioinformatics tools available at the YEASTRACT web page (, [16]). From the 170 transcription factors included in the YEASTRACT database, 32 of them were found to bind to daunorubicin-repressed gene promoters in a significantly higher proportion than expected only by chance (Table 6). The table indicates the total number of genes associated to each transcription factor present in the whole dataset (that is, the 3458 ORF analysed), the number of these genes showing down-regulation by daunorubicin, the expected number by a random distribution (over 280 down regulated genes) and the "enrichment factor", that is, the ratio between observed and expected absolute frequencies for each factor.

**Table 6:** Transcription factors preferently associated to DNR-inhibited genes

Factor	Total regulated genes^a)^	DNR-down regulated genes			*p*	
			
		Observed	Expected (out of 280)	Observed/Expected	Hypergeometric	Bonferroni
Sok2p	561	118	45.45	2.6	5.6 × 10^-27^	7.2 × 10^-25^
Msn2p	316	72	25.58	2.8	2.0 × 10^-17^	2.6 × 10^-15^
Msn4p	286	67	23.13	2.9	8.3 × 10^-17^	1.1 × 10^-14^
Gis1p	91	35	7.35	4.8	1.5 × 10^-16^	1.9 × 10^-14^
Cst6p	104	36	8.44	4.3	4.0 × 10^-15^	5.1 × 10^-13^
Pdr3p	84	29	6.8	4.3	2.4 × 10^-12^	3.1 × 10^-10^
Yap1p	1025	133	83	1.6	2.1 × 10^-11^	2.8 × 10^-9^
Met4p	746	105	60.42	1.7	8.8 × 10^-11^	1.1 × 10^-8^
Adr1p	148	36	11.97	3.0	3.6 × 10^-10^	4.6 × 10^-8^
Xbp1p	84	26	6.8	3.8	5.3 × 10^-10^	6.9 × 10^-8^
Rox1p	202	44	16.33	2.7	6.2 × 10^-10^	7.9 × 10^-8^
Aft1p	397	66	32.11	2.1	9.5 × 10^-10^	1.2 × 10^-7^
Crz1p	155	37	12.52	3.0	1.4 × 10^-9^	1.8 × 10^-7^
Pdr1p	205	42	16.6	2.5	3.9 × 10^-9^	5.1 × 10^-7^
Skn7p	215	44	17.42	2.5	5.4 × 10^-9^	7.0 × 10^-7^
Gcn4p	309	54	25.04	2.2	7.8 × 10^-9^	1.0 × 10^-6^
Stp2p	131	32	10.61	3.0	1.5 × 10^-8^	2.0 × 10^-6^
Hsf1p	266	48	21.5	2.2	5.2 × 10^-8^	6.7 × 10^-6^
Mig1p	74	21	5.99	3.5	1.1 × 10^-7^	1.4 × 10^-5^
Ino2p	81	22	6.53	3.4	1.2 × 10^-7^	1.6 × 10^-5^
Gcr2p	97	25	7.89	3.2	2.8 × 10^-7^	3.6 × 10^-5^
Mga1p	151	31	12.25	2.5	4.6 × 10^-7^	5.9 × 10^-5^
Mbp1p	242	42	19.59	2.1	4.6 × 10^-7^	5.9 × 10^-5^
Rfx1p	87	23	7.08	3.2	6.0 × 10^-7^	7.7 × 10^-5^
Stp1p	91	23	7.35	3.1	1.1 × 10^-6^	1.4 × 10^-4^
Rtg3p	108	24	8.71	2.8	1.9 × 10^-6^	2.4 × 10^-4^
Swi4p	302	47	24.49	1.9	2.5 × 10^-6^	3.3 × 10^-4^
Rgt1p	44	14	3.54	4.0	2.9 × 10^-6^	3.7 × 10^-4^
Ino4p	333	50	26.94	1.9	3.1 × 10^-6^	4.0 × 10^-4^
Sut1p	34	12	2.72	4.4	4.1 × 10^-6^	5.3 × 10^-4^
Gat4p	64	18	5.17	3.5	4.5 × 10^-6^	5.8 × 10^-4^
Nrg1p	168	31	13.61	2.3	4.7 × 10^-6^	6.1 × 10^-4^

a) Number of genes associated to each factor, following YEASTRACT. Only genes used in the microarray analysis (3458) were considered.

Some of these factors (Yap1p, Msn2p, Msn4p) are intimately related to stress response, whereas others, such as Gcr2p, Adr1p, Mig1p and Rgt1p, are associated to carbohydrate and alcohol metabolism. In addition, Gcn4p and Met4p are known regulators of amino acids biosynthetic pathways. In this regard, the transcription factor list recapitulates the functional distribution of daunorubicin down regulated genes in Table 3. Fourteen transcription factors showed enrichment factors over 3 fold, indicating that their associated genes were found in the daunorubicin down regulated dataset at 3 to 5 times higher frequencies than expected (Table 7). Many of these factors are known regulators of glycolytic genes, such as Rgt1p, Mig1p, Gcr2p or Adr1p; therefore, their inclusion in the list may merely reflect the general decrease of transcription of the regulated genes. In addition, this list includes a strikingly high proportion (10 out 14) of transcription factors encompassing CpG steps in their DNA binding sites, irrespectively their relationship with the glycolytic pathway. This observation is consistent with a preferential effect of daunorubicin on the expression of genes regulated by transcription factors with CpG steps in their DNA recognition sequences, in keeping with previous results [8]. This specific inhibition of transcriptional activation by daunorubicin suggests that it may compete with some transcription factors for DNA binding in CpG-reach sequences in gene promoters.

**Table 7:** Transcription factors selectively enriched in daunorubicin-down regulated gene promoters

Factor	Found/expected	*p^a)^*	Binding sequences	CpG steps	Characteristics/Function
Gis1p	4.76	1.9 × 10^-14^	TWAGGGAT, AGGGG		JmjC domain-containing histone demethylase; transcription factor involved in the expression of genes during nutrient limitation; also involved in the negative regulation of DPP1 and PHR1
Sut1p	4.41	5.3 × 10^-4^	CGCG	*	Transcription factor of the Zn [II]2Cys6 family involved in sterol uptake; involved in induction of hypoxic gene expression
Cst6p	4.27	5.1 × 10^-13^	TGACGTCA, TTACGTAA	*	Basic leucine zipper (bZIP) transcription factor of the ATF/CREB family, activates transcription of genes involved in utilization of non-optimal carbon sources; involved in telomere maintenance
Pdr3p	4.26	3.1 × 10^-10^	TCCGCGGA	*	Transcriptional activator of the pleiotropic drug resistance network, regulates expression of ATP-binding cassette (ABC) transporters through binding to cis-acting sites known as PDREs (PDR responsive elements)
Rgt1p	3.95	3.7 × 10^-4^	CGGANNA	*	Glucose-responsive transcription factor that regulates expression of several glucose transporter (HXT) genes in response to glucose; binds to promoters and acts both as a transcriptional activator and repressor
Xbp1p	3.82	6.9 × 10^-8^	GCCTCGARMGA	*	Transcriptional repressor that binds to promoter sequences of the cyclin genes, CYS3, and SMF2; expression is induced by stress or starvation during mitosis, and late in meiosis; member of the Swi4p/Mbp1p family; potential Cdc28p substrate
Mig1p	3.51	1.4 × 10^-5^	W(4-5)GCGGGG	*	Transcription factor involved in glucose repression; sequence specific DNA binding protein containing two Cys2His2 zinc finger motifs; regulated by the SNF1 kinase and the GLC7 phosphatase
Gat4p	3.48	5.8 × 10^-4^	GATA		Protein containing GATA family zinc finger motifs
Ino2p	3.37	1.6 × 10^-5^	WYTTCAYRTGS	*	Component of the heteromeric Ino2p/Ino4p basic helix-loop-helix transcription activator that binds inositol/choline-responsive elements (ICREs), required for derepression of phospholipid biosynthetic genes in response to inositol depletion
Rfx1p	3.25	7.7 × 10^-5^	TCRYYRYRGCAAC	*	Protein involved in DNA damage and replication checkpoint pathway; recruits repressors Tup1p and Cyc8p to promoters of DNA damage-inducible genes; similar to a family of mammalian DNA binding RFX1-4 proteins
Gcr2p	3.17	3.6 × 10^-5^	CTTCC, CWTCC (Gcr1p)		Transcriptional activator of genes involved in glycolysis; interacts and functions with the DNA binding protein Gcr1p
Stp1p	3.13	1.4 × 10^-4^	CGGCN(6)CGGC	*	Transcription factor, activated by proteolytic processing in response to signals from the SPS sensor system for external amino acids; activates transcription of amino acid permease genes and may have a role in tRNA processing
Stp2p	3.02	2.0 × 10^-6^	CGGGGTGN(7)CGCACCG	*	Transcription factor, activated by proteolytic processing in response to signals from the SPS sensor system for external amino acids; activates transcription of amino acid permease genes
Adr1p	3.01	4.6 × 10^-8^	TTGGRGN(6-38)CYCCAA		Carbon source-responsive zinc-finger transcription factor, required for transcription of the glucose-repressed gene ADH2, of peroxisomal protein genes, and of genes required for ethanol, glycerol, and fatty acid utilization

a) Hypergeometric distribution with Bonferroni correction

### Correlation of daunorubicin effects and deletions of transcription factor genes

**Figure 5:**
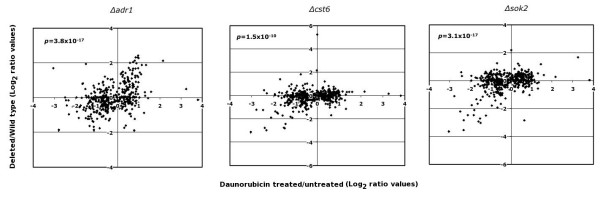
**Transcription ratios between daunorubicin-treated cells and three strains deleted for different transcription factors.** The X-axis corresponds to microarray data for cells treated with daunorubicin for four hours (treated vs. untreated, log_2 _values). The Y-axis corresponds to data from reference [17]. Only data for the 475 genes affected by daunorubicin were considered.

A direct prediction of the DNA-binding competition model for daunorubicin action is that its presence in the cell should produce a phenocopy of genetic deletion of these factors [12], or their partial depletion [7]. To test this prediction, we compared the effects of daunorubicin shown here with a large dataset of null deletions of 42 transcription factors, many of them coincident with the set in Table 6[17]. Table 8 shows the correlation between microarray data from six deletion strains [17] and the corresponding figures from the 4 h daunorubicin-treatment dataset. For these calculations, ratios between deleted and wild type strains were compared to 4 h to 0 h ratios, only for those genes that showed significant variations in expression (positive or negative) due to daunorubicin treatment. The six strains shown in Table 8 are the only ones in the dataset [17] showing positive and significant correlation (p < 0.001, Bonferroni) with daunorubicin-treatment data. The best correlation values corresponded to three strains deleted for factors Adr1p, Cst6p and Sok2p; graphs in Figure 5 show expression ratios for these three strains plotted against the corresponding values from daunorubicin treatment. These plots strongly suggest that at least part of the changes in transcription ratios induced by daunorubicin may be due to competition of the drug with these and other transcription factors for binding to consensus DNA sequences.

**Table 8:** Correlation coefficient and associated *p *values between daunorubicin-treated and Transcription-factor deleted strains^a)^

Deletion strain	r	*p *(T-test)	Bonferroni
Δ*sok2*	0.428	3.1 × 10^-19^	3.1 × 10^-17^
Δ*adr1*	0.427	3.8 × 10^-19^	3.8 × 10^-17^
Δ*cst6*	0.344	1.5 × 10^-12^	1.5 × 10^-10^
Δ*pho4*	0.256	2.1 × 10^-7^	2.1 × 10^-5^
Δ*ste12*	0.239	1.3 × 10^-6^	1.3 × 10^-4^
Δ*hap4*	0.236	1.9 × 10^-6^	1.9 × 10^-4^

a) Only genes significantly altered by daunorubicin treatment were considered (n = 445).

## Discussion

The yeast *Saccharomyces cerevisiae *is a favourite tool for testing drugs that interact and/or modify gene regulation, since it shares many common regulatory mechanisms with vertebrates, ranging from cell cycle to transcriptional regulation [13,18-20]. In a previous paper [12], we showed that daunorubicin specifically inhibited genes required for galactose utilisation, a phenotype we proposed linked to the presence of CpG steps in the recognition sequence of the main regulator for these genes, Gal4p. Here we extended these studies to the whole yeast transcriptome, in conditions of mild inhibition of cell growth.

**Figure 6:**
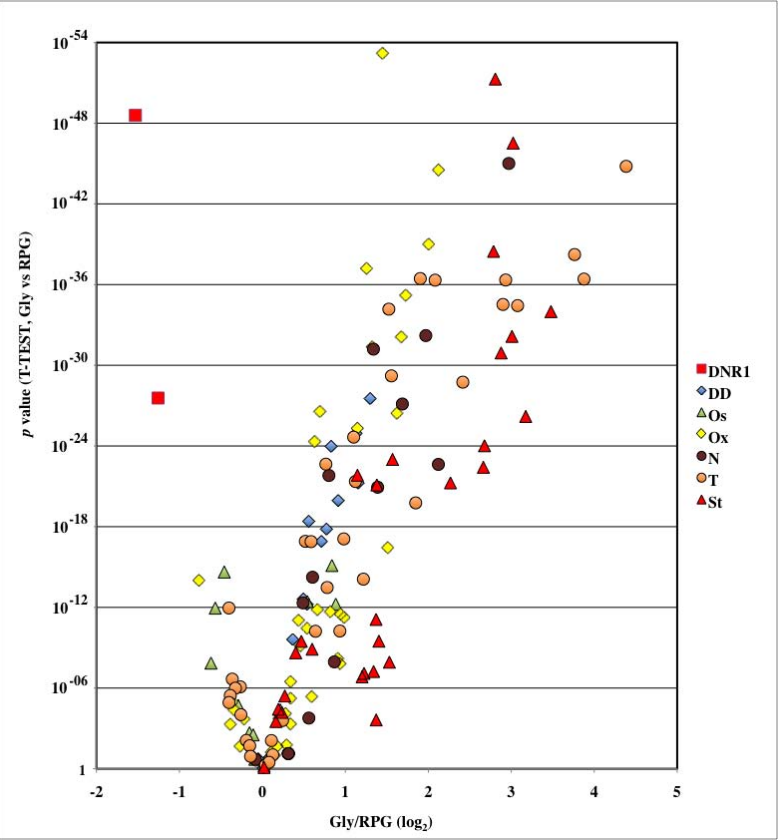
**Differential expression for glycolytic genes (gly) and ribosomal protein genes (rpg) in yeast cells subjected to different treatments.** Fold induction or repression values were calculated for 32 glycolytic genes and 123 ribosomal protein genes for each of the 146 stress conditions, plus the two daunorubicin treatments. The X-axis values correspond to ratios between the average of fold induction/repression for glycolitic and ribosomal protein genes for in each experiment; Y-axis indicates the probability of both sets of genes being equally affected by each treatment. Note the reverse scale of the Y-axis. Each dot represent a single stress dataset for a particular stress condition; they are grouped in several categories: Daunorubicin treatment (DNR, 1 h and 4 h, red squares), DNA damaging agents (DD, 15 conditions, blue diamonds), osmotic stress (OS, 12 conditions, green triangles), oxidative stress (Ox, 45 conditions, yellow diamond), temperature stress (T, 37 conditions, orange circle), amino acid and nitrogen starvation (N, 15 conditions, dark brown circle) and maintenance in stationary phase for long periods of time (22 conditions, red triangles). Two vertical, discontinuous lines indicate 2-fold induction or repression; note that ratio values are expressed as log_2 _transformants. Except for daunorubicin-treatment, all data are from references [21,22]. Genes and conditions analysed are listed in Table 9.

Daunorubicin treatment affected transcription of a relative small proportion of genes. We chose a relatively mild treatment, slightly under the IC_50_, in order to minimise general toxic effects in cell membranes and widespread DNA damage. A conclusion from our analysis is the selective repression by daunorubicin of genes involved in the glycolytic pathway, whereas other genes involved in growth, like ribosomal protein genes, were either not affected or slightly activated. This pattern is very rarely observed in yeast, as glucose utilisation is required for fast growth. Figure 6 shows ratios of expression changes for 32 glycolysis-related genes (gly genes) and 123 ribosomal protein genes (rpg genes) in 146 stress conditions, including DNA damage (both chemical and by irradiation), oxidative and osmotic stress, amino acid and nitrogen starvation, entering in stationary phase, and temperature shifts ([21,22]; list of genes and conditions in Table 9). The graph shows both the ratio between both sets of genes and p-values associated to their differential response to each stress. Low p-values (upper part of the graph, note the reversed Y-axis) correspond to data sets in which the response of both sets of genes showed little or no overlap, whereas high p-values (lower part of the graph) implicate that both sets of genes responded similarly to that specific stress condition. The graph shows that ribosomal protein genes are preferentially inhibited in many stress conditions compared to glycolysis-related genes (right portion of the graph), whereas daunorubicin treatment datasets (1 h and 4 h) differentiate clearly from the rest by specifically depressing glycolytic gene transcription without a parallel decrease of ribosomal synthesis (upper left part of the graph). We concluded that daunorubicin effects couldn't be ascribed to any of the tested stresses, including DNA damage and oxidative stress. This conclusion is further supported by the fact that many stress-related genes, like HSPs, were down regulated, rather than up regulated upon daunorubicin treatment.

**Table 9:** Genes and conditions used for the graph in Figure 6.

Gly genes	rpg genes	rpg genes	Experiments/conditions		
*ADH1*	*RPL10*	*RPL6A*	**DNA damage^a^**	**Osmotic stress^b^**	**Oxidative stress^b^**
*ADH2*	*RPL11A*	*RPL6B*	DES460 + 0.02% MMS - 120 min	1M sorbitol - 120 min	1 mM Menadione (10 min)redo
*ADH3*	*RPL11B*	*RPL7A*	DES460 + 0.02% MMS - 15 min	1M sorbitol - 15 min	1 mM Menadione (105 min) redo
*ADH5*	*RPL12A*	*RPL7B*	DES460 + 0.02% MMS - 30 min	1M sorbitol - 30 min	1 mM Menadione (120 min)redo
*CDC19*	*RPL12B*	*RPL8A*	DES460 + 0.02% MMS - 5 min	1M sorbitol - 45 min	1 mM Menadione (160 min) redo
*ENO1*	*RPL13A*	*RPL8B*	DES460 + 0.02% MMS - 60 min	1M sorbitol - 5 min	1 mM Menadione (20 min) redo
*ENO2*	*RPL13B*	*RPL9A*	DES460 + 0.02% MMS - 90 min	1M sorbitol - 60 min	1 mM Menadione (30 min) redo
*FBA1*	*RPL14B*	*RPL9B*	DES460 + 0.2% MMS - 45 min	1M sorbitol - 90 min	1 mM Menadione (50 min)redo
*GLK1*	*RPL15B*	*RPS0A*	wt_plus_gamma_10_min	Hypo-osmotic shock - 15 min	1 mM Menadione (80 min) redo
*GPM1*	*RPL16A*	*RPS0B*	wt_plus_gamma_120_min	Hypo-osmotic shock - 30 min	1.5 mM diamide (10 min)
*GPM2*	*RPL16B*	*RPS10A*	wt_plus_gamma_20_min	Hypo-osmotic shock - 45 min	1.5 mM diamide (20 min)
*GPM3*	*RPL17A*	*RPS10B*	wt_plus_gamma_30_min	Hypo-osmotic shock - 5 min	1.5 mM diamide (30 min)
*HXK1*	*RPL17B*	*RPS11A*	wt_plus_gamma_45_min	Hypo-osmotic shock - 60 min	1.5 mM diamide (40 min)
*HXK2*	*RPL18A*	*RPS11B*	wt_plus_gamma_5_min		1.5 mM diamide (5 min)
*LAT1*	*RPL18B*	*RPS12*	wt_plus_gamma_60_min	**AA/N starvation^b^**	1.5 mM diamide (50 min)
*PDA1*	*RPL19A*	*RPS13*	wt_plus_gamma_90_min	aa starv 0.5 h	1.5 mM diamide (60 min)
*PDB1*	*RPL19B*	*RPS14A*		aa starv 1 h	1.5 mM diamide (90 min)
*PDC1*	*RPL1A*	*RPS14B*		aa starv 2 h	1 mM Menadione (40 min) redo
*PDC5*	*RPL1B*	*RPS15*	**Temperature^b^**	aa starv 4 h	2.5 mM DTT 005 min dtt-1
*PDX1*	*RPL20A*	*RPS16A*	17 deg growth ct-1	aa starv 6 h	2.5 mM DTT 015 min dtt-1
*PFK1*	*RPL20B*	*RPS16B*	21 deg growth ct-1	Nitrogen Depletion 1 d	2.5 mM DTT 030 min dtt-1
*PFK2*	*RPL21A*	*RPS17A*	25 deg growth ct-1	Nitrogen Depletion 1 h	2.5 mM DTT 045 min dtt-1
*PGI1*	*RPL21B*	*RPS17B*	29 deg growth ct-1	Nitrogen Depletion 12 h	2.5 mM DTT 060 min dtt-1
*PGK1*	*RPL22A*	*RPS18A*	29C to 33C - 15 minutes	Nitrogen Depletion 2 d	2.5 mM DTT 090 min dtt-1
*PGM1*	*RPL22B*	*RPS18B*	29C to 33C - 30 minutes	Nitrogen Depletion 2 h	2.5 mM DTT 120 min dtt-1
*PGM2*	*RPL23A*	*RPS19A*	29C to 33C - 5 minutes	Nitrogen Depletion 3 d	2.5 mM DTT 180 min dtt-1
*STO1*	*RPL23B*	*RPS19B*	33C vs. 30C - 90 minutes	Nitrogen Depletion 30 min.	constant 0.32 mM H2O2 (10 min) redo
*TDH1*	*RPL24A*	*RPS1A*	37 deg growth ct-1	Nitrogen Depletion 4 h	constant 0.32 mM H2O2 (100 min) redo
*TDH2*	*RPL24B*	*RPS1B*	DBY7286 37 degree heat - 20 min	Nitrogen Depletion 5 d	constant 0.32 mM H2O2 (120 min) redo
*TDH3*	*RPL25*	*RPS2*	DBYmsn2/4 (real strain) + 37 degrees (20 min)	Nitrogen Depletion 8 h	constant 0.32 mM H2O2 (160 min) redo
*TPI1*	*RPL26A*	*RPS20*	DBYmsn2-4- 37 degree heat - 20 min		constant 0.32 mM H2O2 (20 min) redo
*TYE7*	*RPL26B*	*RPS21A*	Heat Shock 005 minutes hs-2	**Stationary phase^b^**	constant 0.32 mM H2O2 (30 min) redo
	*RPL27A*	*RPS22A*	Heat Shock 015 minutes hs-2	YPD 1 d ypd-2	constant 0.32 mM H2O2 (40 min) rescan
	*RPL27B*	*RPS22B*	Heat Shock 030inutes hs-2	YPD 10 h ypd-2	constant 0.32 mM H2O2 (50 min) redo
	*RPL28*	*RPS23A*	Heat Shock 05 minutes hs-1	YPD 12 h ypd-2	constant 0.32 mM H2O2 (60 min) redo
	*RPL2A*	*RPS23B*	Heat Shock 060 minutes hs-2	YPD 2 d ypd-2	constant 0.32 mM H2O2 (80 min) redo
	*RPL3*	*RPS24A*	Heat Shock 10 minutes hs-1	YPD 2 h ypd-2	DBY7286 + 0.3 mM H2O2 (20 min)
	*RPL30*	*RPS24B*	Heat Shock 15 minutes hs-1	YPD 3 d ypd-2	DBYmsn2/4 (real strain) + 0.32 mM H2O2 (20 min)
	*RPL31A*	*RPS25A*	heat shock 17 to 37, 20 minutes	YPD 4 h ypd-2	DBYmsn2msn4 (good strain) + 0.32 mM H2O2
	*RPL31B*	*RPS25B*	Heat Shock 20 minutes hs-1	YPD 5 d ypd-2	dtt 000 min dtt-2
	*RPL32*	*RPS26A*	heat shock 21 to 37, 20 minutes	YPD 6 h ypd-2	dtt 015 min dtt-2
	*RPL33A*	*RPS26B*	heat shock 25 to 37, 20 minutes	YPD 8 h ypd-2	dtt 030 min dtt-2
	*RPL33B*	*RPS27A*	heat shock 29 to 37, 20 minutes	YPD stationary phase 1 d ypd-1	dtt 060 min dtt-2
	*RPL34B*	*RPS27B*	Heat Shock 30 minutes hs-1	YPD stationary phase 12 h ypd-1	dtt 120 min dtt-2
	*RPL35A*	*RPS28A*	heat shock 33 to 37, 20 minutes	YPD stationary phase 13 d ypd-1	dtt 240 min dtt-2
	*RPL35B*	*RPS28B*	Heat Shock 40 minutes hs-1	YPD stationary phase 2 d ypd-1	dtt 480 min dtt-2
	*RPL36A*	*RPS29A*	Heat Shock 60 minutes hs-1	YPD stationary phase 2 h ypd-1	
	*RPL37A*	*RPS29B*	Heat Shock 80 minutes hs-1	YPD stationary phase 22 d ypd-1	
	*RPL37B*	*RPS3*	steady state 15 dec C ct-2	YPD stationary phase 28 d ypd-1	
	*RPL38*	*RPS30A*	steady state 17 dec C ct-2	YPD stationary phase 3 d ypd-1	
	*RPL39*	*RPS30B*	steady state 21 dec C ct-2	YPD stationary phase 4 h ypd-1	
	*RPL40A*	*RPS31*	steady state 25 dec C ct-2	YPD stationary phase 5 d ypd-1	
	*RPL40B*	*RPS4A*	steady state 29 dec C ct-2	YPD stationary phase 7 d ypd-1	
	*RPL41A*	*RPS4B*	steady state 33 dec C ct-2	YPD stationary phase 8 h ypd-1	
	*RPL42A*	*RPS6A*	steady state 36 dec C ct-2		
	*RPL42B*	*RPS6B*	steady state 36 dec C ct-2 (repeat hyb)		
	*RPL43A*	*RPS7A*			
	*RPL43B*	*RPS7B*			
	*RPL4A*	*RPS8A*			
	*RPL4B*	*RPS8B*			
	*RPL5*	*RPS9A*			
		*RPS9B*			

a) Data from reference [21]

b) Data from reference [22]

Inspection of promoters of daunorubicin-inhibited genes showed that they present a significant high proportion of DNA binding sites for a defined subset of transcription factors, most of them related to sugar metabolism. These data have to be interpreted not necessarily as an indication of direct interaction of the drug with these transcription factors, but only as a hint of the regulatory networks, or regulons, particularly affected by the drug. Due to the complexity of eukaryotic promoters, several factors may appear in any particular affected promoter, although the putative direct effect of the drug may affect to only one or two of them. A particularly relevant example is Mig1p, a transcriptional repressor central in the catabolite repression by glucose and that binds to many glycolytic gene promoters [23]. Therefore, it appears on the lists of transcription factors preferentially associated to daunorubicin-inhibited genes (Tables 6 and 7), although the hypothetical suppression of its binding to DNA would result in activation, rather than inhibition, of the affected gene. This is the most reasonable explanation by the appearance in these lists of some transcription factors that do not encompass daunorubicin-preferred sites in their recognition sequences (Table 7).

Data mining identified several microarray datasets with patterns resembling to the ones observed in daunorubicin-treated cells. Best correlations were observed for strains deleted for some glucose-related transcription factor genes, especially *ADR1*, *CST6 *and *SOK2*. Deletion of these genes results in a general decrease on transcription of glycolytic genes with relatively mild effects on transcription of genes related to cell growth, like ribosomal protein genes -exactly the pattern observed in daunorubicin-treated cells. Two of these three factors (Adr1p and Cst6p) were identified as preferentially associated to genes down regulated by daunorubicin (Table 6, Figure 4). This list also includes a high proportion of factors whose DNA recognition sequences include CpG steps, the preferred binding site for daunorubicin [4]. Therefore, we concluded that daunorubicin inhibition of yeast growth might be mediated by its interaction with DNA at sequences also recognized by some transcription factors, resulting in a transcriptional repression of glycolytic genes, among others. These results corroborate the interest in using yeast mutants as an *in vivo *system to identify the determinants of chemosensitivity [13].

The amazing conservation of regulatory elements among opisthokonta (taxon that includes fungi and animals, among other groups) allows identification of pathways and transcription factors common to yeast and humans. For example, Cst6p is a basic leucine zipper transcription factor of the ATF/CREB family, which includes *bona fide *orthologues in mammals, not only in functional terms (targets for the cAMP regulatory pathway), but also by their binding to identical DNA sequences, 5'-TGACGTCA-3' [24]. This sequence includes a high affinity site for daunorubicin, providing an explanation for several of the effects observed in this work. Sok2p is also known to participate in the cAMP regulatory pathway [25], and, therefore, many cAMP-regulated promoters encompass binding sites for both factors. This circumstance provides a good explanation for the good correlation between the changes in gene expression due to the deletion of the corresponding gene and those observed upon daunorubicin treatment, although the DNA recognition sequence for Sok2p (5'-TGCAGNNA-3', [26]) does not include high affinity sites for daunorubicin. Therefore, our data suggest that daunorubicin may target the cAMP signalling pathway of yeast, inhibiting expression of many regulated genes and particularly those under control of Cst6p, ant that may be explained by binding of the drug to the Cst6p DNA recognition site. The question of whether daunorubicin may have similar effects in the cAMP-mediated regulation of proliferation of mammalian cells is still open.

Extrapolation of these results to tumour cells can be undertaken at several levels. First, as a general model, they demonstrate that DNA-intercalating drugs can block cell growth by selectively reducing the efficiency of different transcription factors. If these factors are required for cell growth, this would prevent tumour propagation at effective concentration of the drug much below the ones required for the massive DNA damage required to trigger apoptosis [27,28]. In addition, the specific effects of daunorubicin on the glycolysis pathway may be relevant to its antitumor effect. One of the most outstanding alterations in cancer cells is their dependence on glycolytic pathways for the generation of ATP [29], and there is compelling evidence that mitochondrial defects in tumour cells under hypoxia are remarkably sensitive to glycolysis inhibition [29]. Besides, it has been recently reported that some inhibitors of glucose uptake sensitize tumour cells to daunorubicin [30]. Our data would suggest that daunorubicin might work not only as a DNA-damaging agent but also as an inhibitor of glycolytic pathways, a combined effect that might have broad therapeutic implications against cancer cells growing under hypoxic conditions.

## Conclusion

The yeast *Saccharomyces cerevisiae *is a powerful tool for the study the effects of drugs on eukaryotic cells. We showed that the antitumor drug daunorubicin alters transcription of some very specific subsets of genes, in a pattern in which sugar- metabolising pathways become down-regulated whereas proliferation-related genes, like ribosomal protein genes, are unaffected or even activated. This pattern is very similar to the one observed in yeast strains deleted for some transcription factors related to the regulation of the glycolytic pathway, like Adr1p, Cst6p and Sok2p. This results are consistent with the hypothesis that daunorubicin impairs binding of different transcription factors by competing for their DNA binding sequences, therefore limiting their effectiveness and affecting the corresponding regulatory networks. This proposed mechanism might have broad therapeutic implications in cancer therapeutics.

## Methods

### Yeast growth and daunorubicin treatment

Daunorubicin (Sigma, St. Louis, MO, U.S.A.) was freshly prepared as a 2 mM stock solution in sterile 150 mM NaCl solution, and diluted to each final concentrations before use. A single colony of *S. cerevisiae *(BY4741 *erg6Δ *(MAT**a**, *his3Δ1, leu2Δ0, met15Δ0, ura3Δ0*, YML008c::KanMX4, from EUROSCARF, Frankfurt, Germany) was inoculated into 25 ml of YPD medium (10 g/L yeast extract, 20 g/L peptone and 20 g/L dextrose) and grown overnight at 30°C in an environmental shaker (250 rpm) until exponential phase. This yeast culture was used to inoculate 500 ml of YPD to an initial A_600 _of 0.1 and further incubated at the same conditions until A_600 _= 0.4. This culture was then divided into three aliquots and diluted four times with fresh YPD medium. Daunorubicin was then added to each culture at a final concentration of 12 mM and cultures were allowed to grow for 1 or 4 hours. The whole procedure was repeated for Real-Time quantitative PCR (qRT-PCR) validation; in this case, only two biological replicas were obtained.

### RNA Preparation

Cultures were centrifuged for 5 min at 3000 rpm, washed with 5 ml MilliQ water and subsequently centrifuged (repeated twice). Total RNA was extracted with the RiboPure Yeast kit (Ambion, Austin, TX, USA). Total RNA was quantified by spectrophotometry in a NanoDrop ND-1000 (NanoDrop Technologies, Wilmintong DE, USA) and its integrity checked on TBE-agarose gels. The resulting total RNA was then treated with DNAseI I (F. Hoffmann-La Roche, Basel Switzerland) to remove contaminating genomic DNA.

### DNA Microarray Analysis

Microarrays used in this work were produced at the Genomics Unit of the Scientific Park of Madrid (Spain). They consist of 13,824 spots, each one corresponding to a synthetic oligonucleotide (70-mer, Yeast Genome Oligo Set, OPERON, Cologne, Germany) encompassing the complete set of 6306 ORFs coded by the *S. cerevisiae *genome. Each ORF was printed at least twice; 600 spots were used as negative controls, either void or printed with random oligonucleotides; a small subset of genes (*ACT1*, *HSP104*, *NUP159*, *NUP82*, *RPL32*, *RPS6B*, *SWI1*, *TDH1*, *TDH2*, *TUB4 *and *UBI1*) were printed between 6 and 12 times for testing reproducibility.

Fifteen *μ*g of total RNA were used for cDNA synthesis and labelling with Cy3-dUTP and Cy5-dUTP fluorescent nucleotides, following indirect labelling protocol (CyScribe post-labelling kit, GE-Healthcare, New York, NY, USA). Labelling efficiency was evaluated by measuring Cy3 or Cy5 absorbance in Nanodrop Spectrophotometer. Microarray prehybridization was performed in 5× SSC (SSC: 150 mM NaCl, 15 mM Na-citrate, pH 7.0), 0.1% SDS, 1%BSA at 42°C for 45 min. (Fluka, Sigma-Aldrich, Buchs SG, Switzerland). Labelled cDNA was dried in a vacuum trap and used as probe after resuspension in 110 *μ*l of hybridization solution (50% Formamide, 5×SSC, 0.1% SDS, 100 *μ*g/ml salmon sperm from Invitrogen, Carlsbad, CA, USA). Hybridization and washing were performed in a Lucidea Slide Pro System (GE Healthcare, Uppsala, Sweden). Arrays were scanned with a GenePix 4000B fluorescence scanner and analyzed by Genepix 5.0 Pro software (Axon Instruments, MDS Analytical Technologies, Toronto, Canada). Data was filtered according to spot quality. Only those spots whose intensity was twice background signal and, at least 75% of pixels had intensities above background plus two standard deviations were selected for further calculations. In average, about 60 to 70% of spots in each array were considered suitable for further analysis following these criteria.

### Quantitative Real Time RT-PCR Assay

An aliquot of RNA preparations from untreated and treated samples, used in the microarray experiments, was saved for qRT-PCR follow-up studies. First strand cDNA was synthesized from 2 *μ*g of total DNAseI-treated RNA in a 20 *μ*l reaction volume using Omniscript RT Kit (Qiagen, Valencia, CA, USA) following manufacture's instructions. qRT-PCR reactions were performed by triplicate using the ABI-PRISM 7000 Sequence Detection System (Applied Biosystems, Foster City, CA, USA) using the SYBR Green PCR Master Mix (Applied Biosystems). Gene-specific primers (listed in Table 4) were designed using Primer Express software (Applied Biosystems). Amplified fragments were confirmed by sequencing in a 3730 DNA Analyzer (Applied Biosystems) and sequences were compared with the published genomic data at SGD. Real time PCR conditions included an initial denaturation step at 95°C for 10 min, followed by 40 cycles of a two steps amplification protocol: denaturation at 95°C for 15 s and annealing/extension at 60°C for 1 min. Relative expression values of different genes were calculated following the ΔΔ*C*_*T *_method [31,32], using *RPO21 *as reference gene.

### Clustering and statistical analysis

Our experimental design allowed to obtain up to 6 determinations for each gene and condition: three biological replicates per condition, two replicated spots for each gene in the array. Statistical analyses only considered genes for which a minimum of nine (out of 18) data values passed the microarray quality standards (3458 genes). Data were calculated as binary logarithms (log_2_) of fluorescence ratios (treated versus untreated samples). Significant changes on expression values between the starting point (time 0) and samples taken at 1 and 4 hours of daunorubicin treatment were determined by the Student's T-test. The whole dataset, combining data from the three time points, was analyzed with the TIGR MeV program [33]. Data were normalised by experiments and clustered by hierarchical clustering (Euclidean distance), treating duplicated spots as independent data series. Genes showing significant variations between time points were identified by ANOVA with the Bonferroni correction (p < 0.05). These genes were grouped by their expression patterns in a two-dimensional map grid by SOM (Self-Organizing Maps) [34], to generate hypotheses on the relationships and the function of genes. Classification of genes by gene ontology (GO) in biological process categories [35] was performed in the SDG page. Documented regulators of both affected and non-affected genes were retrieved from YEASTRACT [16]. Statistical analyses on the frequency of regulated genes in different subsets of data were performed using hypergeometric distribution tests with the Bonferroni correction (see SGD page, and )

## Authors' contributions

MR: Growth effects, microarray analysis, qRT-PCR. MC: qRT-PCR analysis, technical assistance. JP & BP: co-direction, data mining and analysis, preparation and writing of the manuscript. All co-authors read and approved the manuscript.
